# Identification of New Natural Sources of Flavour and Aroma Metabolites from Solid-State Fermentation of Agro-Industrial By-Products

**DOI:** 10.3390/metabo12020157

**Published:** 2022-02-08

**Authors:** Melodie A. Lindsay, Ninna Granucci, David R. Greenwood, Silas G. Villas-Boas

**Affiliations:** 1 School of Biological Sciences, University of Auckland, 3A Symonds Street, Auckland 1010, New Zealand; melodie.lindsay@auckland.ac.nz (M.A.L.); n.granucci@greenspot-tech.com (N.G.); david.greenwood@auckland.ac.nz (D.R.G.); 2 Luxembourg Institute of Science and Technology, 41, Rue du Brill, L-4422 Luxembourg, Luxembourg

**Keywords:** *Aspergillus niger*, *Aspergillus oryzae*, *Penicillium camenberti*, *Pycnorporus cinnabarinus*, fruit, vegetable

## Abstract

Increasing consumer demand for natural flavours and fragrances has driven up prices and increased pressure on natural resources. A shift in consumer preference towards more sustainable and economical sources of these natural additives and away from synthetic production has encouraged research into alternative supplies of these valuable compounds. Solid-state fermentation processes support the natural production of secondary metabolites, which represents most flavour and aroma compounds, while agro-industrial by-products are a low-value waste stream with a high potential for adding value. Accordingly, four filamentous fungi species with a history of use in the production of fermented foods and food additives were tested to ferment nine different agro-industrial by-products. Hundreds of volatile compounds were produced and identified using headspace (HS) solid-phase microextraction (SPME) coupled to gas chromatography–mass spectrometry (GC–MS). Four compounds of interest, phenylacetaldehyde, methyl benzoate, 1-octen-3-ol, and phenylethyl alcohol, were extracted and quantified. Preliminary yields were encouraging compared to traditional sources. This, combined with the low-cost substrates and the high-value natural flavours and aromas produced, presents a compelling case for further optimisation of the process.

## 1. Introduction

A high consumer demand for natural flavour and aroma compounds has increased the pressure on traditional, natural sources of many natural additives [1]. Natural flavours and fragrances are traditionally sourced from herbs, spices, plants, and animals—usually in the form of essences, extracts, and oils. The organoleptic properties of such flavours and fragrances tend to be highly complex and difficult to reproduce realistically by synthetic means. As a result, commercial demand for such products often outstrips global supply and prices can be volatile.

Microorganisms have been used to produce and enhance flavours in food and beverage products for centuries, for example, cheese, wine, and chocolate [2,3]. In recent times, there has been a resurgence of using fermentation to produce consumer goods [4]. Various fungi and yeasts have been investigated for their potential to produce aroma compounds, including *Rhizopus* spp., *Trichoderma* spp., *Ceratocystis* spp., *Saccharomyces* spp. And *Hanseniaspora* spp. [5,6,7,8,9,10,11,12].

Solid-state fermentation (SSF) is a promising method to produce natural flavours and fragrances using filamentous fungi, as it offers a complex growth medium that closely mimics the natural environment of these organisms. It is characterised by a fermentation carried out on solid particles, in the absence of free water [13]. Most often, this is a controlled fermentation where either bacteria or yeast grow on the surface of the solid particles in a biofilm, or in the case of filamentous fungi, hyphae can penetrate the solid particles [13]. In such cases, microorganisms tend to produce more complex metabolites—including volatile molecules that contribute to aroma and flavour [14]. SSF has been traditionally used in food fermentation such as during cheese ripening, mushroom production, and part of soy source production.

Many different aroma compounds have been produced through SSF on a range of substrates, including coffee husk, cassava bagasse, apple pomace, wheat bran, citrus pulp, and sugarcane bagasse [5,7,9,10]. This presents an encouraging basis to investigate the potential of a range of agro-industrial by-products for use in SSF to produce flavour and aroma chemicals. In the present study, the volatile profiles of 36 axenic fermentations, using four different filamentous fungi and nine different food-grade agro-industrial by-products, were screened using headspace solid-phase microextraction coupled to gas chromatography–mass spectrometry (HS-SPME-GC/MS). This enabled us to identify potential aroma compounds of commercial interest that can be produced naturally via fermentation and from a sustainable, often wasted, raw material.

## 2. Results

### 2.1. Solid-State Fermentation Performance

Fermentations were visually assessed daily to determine the fermentation end point through substrate colonisation. All substrates were >90% surface covered by mycelia 10 days post-inoculation with the exception of five fermentations: *Aspergillus niger* on red grape marc; *Penicillium camenberti* on olive cake and red grape marc; and *Pycnorporus cinnabarinus*, on spent brewer’s grain, and red grape marc.

### 2.2. Profile of Volatile Compounds

**Figure 1:**
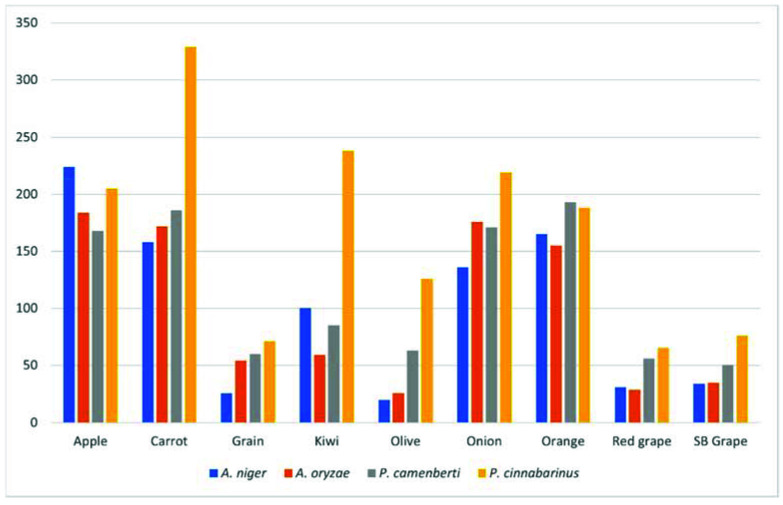
Absolute number of volatile metabolites that increased in abundance in all biological replicates (n = 5) following fermentation with *Aspergillus niger*, *Aspergillus oryzae*, *Penicillium camenberti*, and *Pycnoporus cinnabarinus* on different fruit and vegetable by-products when compared to the unfermented substrate (negative control). Apple = apple pomace, Carrot = carrot pomace, Grain = spent brewer’s grain, Kiwi = kiwifruit skins, Olive = olive cake, Onion = onion pulp, Orange = orange pomace, Red grape = red grape marc, and SB grape = Sauvignon blanc grape marc. Metabolites not detected in all five replicates were excluded from the total.

Hundreds of volatile compounds were detected and putatively identified using a commercial MS library of mass spectra. All substrates produced volatile compounds following fermentation (Figure 1). Over 50 metabolites of interest were identified across the fermented substrates, mostly molecules associated with flavours and aromas. Only analytes with a match factor of over 90% to the NIST library hit in addition to having their level increased after fermentation when compared to the unfermented control were investigated for aroma descriptor qualities. A summary of the topmost abundant compounds that were produced during each fermentation is presented in Table 1 along with their reported aroma descriptor properties.

**Figure 2:**
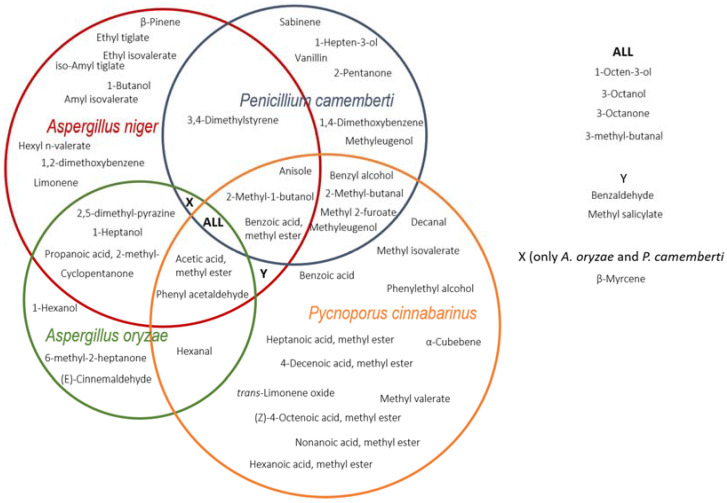
Based upon the volatile metabolite level ratios and statistical significances (*p* value < 0.05), we visualise similarities and differences in metabolite profile among fungal species independent of the substrate fermented. The fold-change values are found in Table 1.

Four compounds were short-listed for identity confirmation, and quantification from the fermented substrates due to their commercial relevance: methyl benzoate, detected only in the headspace of orange pomace fermented by the different fungi except by *Aspergillus oryzae* (Table 1 and Table 2, Figure 2); phenylacetaldehyde, detected in the headspace of different substrates fermented by different fungi (Table 1, Figure 2); 1-octen-3-ol, detected in the headspace of all fermentations (Table 1, Figure 2); and phenylethyl alcohol, only detected in the headspace of brewery spent grain fermented by *P. cinnabarinus* (Table 1 and Table 2, Figure 2). Their fermentation yield and market information are presented in Table 3. While yield was very low for methyl benzoate (0.173 g/kg), higher yields were obtained for 1-octen-3-ol (1.493 g/kg), phenylacetaldehyde (1.297 g/kg) and phenylethyl alcohol (0.970 g/kg). However, while yield is an important factor from a commercial perspective, the premium prices of natural compounds often far surpass that of their synthetic alternatives. In that light, identifying potential valuable products in our fermentations was an important first step of investigating their potential production from fruit and vegetable by-products.

## 3. Discussion

Thousands of tonnes of phenylethyl alcohol are consumed across the food, beverage, cosmetics, and fragrance industries every year. Most of this is synthetically produced from benzene, styrene, or toluene due to the extremely low yield of natural product from its traditional source—rose petals [15,16]. The yield of rose essential oil from rose petals is 0.03–0.04% and comprises up to 60% phenylethyl alcohol [15,17]. Our yield was significantly higher than this; moreover, spent brewer’s grain is a cheap substrate (~USD 300/tonne), while rose petals cost upwards of USD 3000/tonne. With further optimisation, the fermentative production of phenylethyl alcohol from grain could be a competitive, natural source of this molecule.

1-Octen-3-ol was originally discovered in a Japanese mushroom, *Armillaria matsutake* [16,18,19]. Currently, it is synthesised through a reaction between magnesium amyl bromide and acrolein [16]. However, there is demand for natural 1-octen-3-ol as a mushroom flavouring in food products. As a high-value chemical (USD 4800/kg) with over 250 kg used annually in the food and beverage sector alone, our fermentation process could be a good option for the natural production of this metabolite [16]. It is well known that many fungal species produce 1-octen-3-ol as part of their volatilome, including *Penicillium*, *Aspergillus*, and many edible mushroom species [20,21,22,23,24]. Zawirska-Wojtasiak [22] investigated natural 1-octen-3-ol extracted from several edible mushroom varieties finding high optical purity and yields of over 6 mg/100g in various types of edible mushrooms. These could be good candidates to include in further trials using fruit and vegetable by-products to increase and optimise fungal production of this compound.

Phenylacetaldehyde is another volatile compound identified and quantified in fermented spent brewer’s grain that has potential as a natural flavour and fragrance ingredient. It is currently produced chemically from benzaldehyde through a Darzen glycidic ester synthesis [16]. However, it can also be produced through an expensive biotechnological route from phenylalanine and therefore carry a natural label. Phenylacetaldehyde is found as a component of many essential oils and contributes to the flavour of foods such as chocolate, cheeses, coffee, fruits, wine and breads [25,26,27,28,29].

Some of the identified aroma compounds were uniquely associated to a specific substrate (Table 2), whereas the profile of volatile metabolites of some fermented substrates, e.g., onion pulp, red grape marc, Sauvignon blanc grape marc, and kiwifruit skins showed far fewer compounds compared to substrates such as carrot, apple and orange pomaces (Figure 1). As all substrates were used in an unsupplemented state (the only treatment was autoclaving to sterilise the substrates), this leaves the potential to investigate supplementation or fortification of the substrates with nutrients, e.g., nitrogen/phosphorus/potassium and/or sugars. For example, as red grape marc is pre-fermented by yeast and kiwifruit skins are naturally very low in sugar, both substrates may perform better if supplemented with additional sugar. On the other hand, Sauvignon blanc grape marc is high in fermentable sugars; however, it also has a high lignin content, which can be slow to break down. A two-step fermentation using a lignin-degrading fungus such as *P. cinnabarinus* followed by a second fungus could be an option to help increase the available nutrients and produce more of a desired compound [30]. Nonetheless, potentially some unique aroma molecules were found in the headspace of these poorly fermented substrates such as traces of (E)-cinnemaldehyde (cinnamon aroma) in red grape marc fermented by *A. oryzae* and vanillin in olive cake fermented by *P. camemberti*, which are worth further investigation (Table 2).

Among the filamentous fungi used in this study to ferment different fruit and vegetable by-products, *P. cinnabarinus* and *A. niger* showed the best performance regarding the production of volatile compounds (Figure 1). These two fungi also produced the largest number of unique volatile compounds independent of substrate (Figure 2). Comparatively, *A. oryzae* presented the lowest number of unique volatiles, being generally outperformed by the other three fungi regarding their volatilome complexity (Figure 1 and Figure 2). However, only *A. oryzae* was capable of producing cinnamon aroma from red grape marc, albeit in limited amounts. Similarly, *P. camemberti* was the only fungus capable of producing trace amounts of vanillin from olive cake. To the best of our knowledge, this is the first-time vanillin has been detected in *P. camemberti* fermentation, suggesting this fungus is capable of producing this aroma compound from a precursor available in olive cake (Table 1, Figure 2). Many filamentous fungi are capable of biosynthesising vanillin from different phenolic acids, including *P. cinnabarinus* [31,32,33]. However, our strain of *P. cinnabarinus* did not produce this aroma compound in any fermentation. Olive cake is a substrate rich in lignin, polyphenols, and phenolic acids [34,35]. Therefore, *P. camemberti* may have converted some phenolic acids commonly present in olive cake into vanillin, such as ferulic acid and or vanillic acid [35].

Nine different substrates were investigated, of which apple, carrot, and orange pomaces appeared to perform the best, producing the most volatiles. Therefore, any further screening experiments should focus on these substrates where available. Further experiments could also include investigating different fungal strains for their potential to produce high-value 1-octen-3-ol. Many edible mushrooms—especially *Agaricus bisporus*—produce high quantities of 1-octen-3-ol and should be investigated for their potential to ferment agro-industrial by-products such as onion pulp. As onions present high levels of linoleic acid, which is a precursor in the production of 1-octen-3-ol, this could be a very promising combination of substrate and fermenting fungi [36].

Solid-state fermentation is still in its infancy compared with industrial liquid-state fermentations. While there is potential to produce more complex secondary metabolites by using SSF, there are still problems with the large surface area required, air circulation, assuring consistent fermentations, as well as a heightened risk of contamination [14]. There are also several downstream challenges associated with extracting volatile compounds from the fermented substrate. While a distillate or extraction into ethanol could be feasible, distillation and/or purification can be expensive and challenging. Many essential oils and extracts contain mixtures of hundreds of compounds that convey a desired flavour or fragrance that is readily accepted by many consumers. In this study, all the fungi used have a history of use in food fermentation or food ingredient production and the fruit and vegetable by-products were of food-grade quality. Provided the compound of interest is not masked or otherwise negatively affected by the presence of co-produced compounds, there is a good case to bring this compound to market as an essence, extract, or essential oil derived from the fermented substrate. This is the case for many natural fragrance and flavour additives, for example, rose oil, which contains up to 60% phenylethyl alcohol and vanilla extract, which contains only 0.1–0.25% vanillin [17,37,38].

## 4. Materials and Methods

### 4.1. Microorganisms

The fungal strains *Aspergillus niger* ICMP 17511 and *Aspergillus oryzae* ICMP 1281 were obtained from the International Collection of Microorganisms from Plants (ICMP) maintained by Manaaki Whenua Landcare Research (www.landcareresearch.co.nz/ (accessed on 3 February 2022). *Penicillium camenberti* ZX27 was purchased from Mad Millie NZ (Auckland, New Zealand). *Pycnorporus cinnabarinus* SVB-F118 was isolated from bush debris in New Zealand. All fungal strains were maintained on Sabouraud dextrose agar Auckland, New Zealand), Simply Squeezed Limited (Napier, New Zealand), RD2 International Limited (Auckland New Zealand), Onions NZ (Pukekoe, New Zealand), Kiwifruitz (Tauranga, New Zealand), and Pernod Ricard New Zealand (Auckland New Zealand). By-products included apple pomace; orange pomace; carrot pomace; onion pulp; kiwifruit skins; grape marc (red and white); spent brewer’s grain; and olive cake. All substrates were used without nutritional supplementation for all solid-state fermentations. All substrates were stored at −20 °C in vacuum-sealed freezer bags until use in fermentations.

### 4.2. Solid-State Fermentations

Prior to fermentation, each substrate was thawed and sterilised by autoclaving at 121 °C for 20 min. *A. niger*, *A. oryzae* and *P. camenberti* were sporulated on Sabouraud dextrose agar (SDA) medium (Merck KGaA, Darmstadt, Germany). *P. cinnabarinus* was propagated on potato dextrose agar (PDA) medium (Merck KGaA, Darmstadt, Germany) and a preinoculum (myceliated grains) was obtained on sterilised rice cultured at 25 °C until complete colonisation. Five 500 mL Erlenmeyer flasks containing 50 g of sterile substrate without adjusting their moisture content and substrate pH were inoculated with spore suspension (3 mL 10^7^ spores/mL in 0.9% saline solution) or myciliated rice preinoculum (5 g wet weight). The flasks were then incubated at 25 °C (*P. cinnabarinus* and *P. camenberti*) or 28 °C (*A. niger* and *A. oryzae*) in the dark until more than 90% substrate colonisation was reached through visual inspection. Each substrate/fungus combination and corresponding sterile, negative controls were incubated in parallel. Fermentations were visually assessed daily to determine the fermentation end point through substrate colonisation. All substrates were >90% surface covered by mycelia 10 days post-inoculation with the exception of five fermentations: *Aspergillus niger* on red grape marc; *Penicillium camenberti* on olive cake and red grape marc; and *Pycnorporus cinnabarinus*, on spent brewer’s grain, and red grape marc. After fermentation, samples were stored at −20 °C in glass vials flushed with nitrogen pending volatiles analysis.

### 4.3. Headspace Solid-Phase Microextraction (HS-SPME) Coupled to Gas Chromatography–Mass Spectrometry (GC–MS)

Solid-state fermentation samples were thawed overnight at 4 °C, weighed into 20 mL amber SPME headspace vials (2 g wet weight) and immediately fitted with a silicone/PTFE septum cap (Supelco). All extractions were carried out using a 1 cm 50/30 µm DVB/CAR/PDMS (Supelco) fibre. Preincubation (10 min at 60 °C) was immediately followed by extraction (10 min). Desorption in the GC was performed under splitless mode (1 min at 250 °C). Then, the fibre was cleaned (5 min at 250 °C) in preparation for the next sample.

Volatile compounds adsorbed into the SPME fibre were analysed by GC–MS by desorbing them into a Shimadzu QP2010 Plus GC–MS system via a CTC analytics Combi PAL autosampler. A fused silica HP-5MS column (30 m × 250 µm × 0.25 µm) from Agilent was used to separate the analytes. The inlet temperature was fixed at 250 °C. The column flow rate was kept constant at 1 mL/min using ultra-high-purity-grade helium gas. The initial GC oven temperature was set at 35 °C, then immediately increased to 80 °C at a rate of 10 °C/min upon sample injection. Then, ramped again to 160 °C at a rate of 2 °C/min, followed by a final ramp at 10 °C/min to 260 °C. The GC–MS interface temperature was held at 250 °C, the ion source at 200 °C, and quadrupole at 200 °C. The ion source was operated in electron impact ionisation mode at 70 eV. Compounds were detected using mass spectra acquired in scan mode in the range of 33 to 400 *m*/*z*.

### 4.4. SPME-GCMS Data Processing

We used the Automated Mass spectral Deconvolution and Identification System (AMDIS) software to process the SPME data generated by GC–MS. The compounds were identified using the National Institute of Standards and Technology (NIST) 2017 mass spectral library, only considering those with a match quality above 90% (putative identification). The identity of selected compounds was confirmed using authentic standards. An in-house R package was used for automated integration of reference ion peak area. Each identification was individually screened, and manual retention time correction and subsequent re-integration was performed where required. Only compounds that increased in abundance or were produced de novo were considered for further characterisation experiments.

To determine the fold-change increase in volatile metabolites after fermentation, the analyte peak areas were divided by the total ion count (TIC) of the corresponding data file. The peak abundances in proportion to the TIC were subsequently divided by the raw peak area of hexamethyl cyclotrisiloxane as a means of external standard normalisation. The resulting data were log-transformed and Pareto-scaled to make the features more comparable within the data. Student’s t-test was applied to determine whether the relative abundance of compounds detected in a different group of samples (data classes) was significantly different between growth conditions, which was defined as *p*-value < 0.05 after adjustment by false discovery rate (FDR).

### 4.5. Identification Confirmation and Quantitation of Volatile Compounds

Compounds of interest were putatively identified in several fermentations through SPME-GCMS analysis. These fermentations were then repeated and used for extraction of volatile compounds to determine fermentation yield and confirm identification using pure standards. For each extraction, the homogenised sample (2 g) was weighed into a Kimax test tube and distilled water (1 mL), internal standard (5 µL, 12-bromo-dodecanol 10 mM) and sodium chloride (~100 mg) were added. Tetrahydrofuran (1 mL) was aliquoted into each tube, capped, and vigorously mixed using a vortex mixer (2 min). Samples were sonicated for 30 min followed by centrifugation at 4000 rpm for 10 min. The organic phase was aspirated into GC–MS vials and kept at 4 °C in a cooling tray pending GC–MS analysis.

The analytes were quantified using a GC-7890 gas chromatograph (Agilent Technologies, Santa Clara, CA) coupled to an MSD-5975 mass spectrometer (Agilent Technologies, Santa Clara, CA). The column used was a fused silica Rtx^®^-5Sil MS 30 m × 250 μm × 0.25 μm (Restek). The GC–MS parameters used for the SPME analysis described above were kept the same.

The identity of all four compounds was confirmed by comparing their chromato-graphic retention times and mass spectra to those of pure standards. A calibration curve was built for each compound of interest using pure chemical standards obtained from Sigma Aldrich and analysed in the same time as the extracted samples. Each compound was quantified using peak height normalised by internal standard peak height using the calibration curve of pure standard. Calibration curve characteristics are presented in Table A1 (Appendix A). Yield was determined from the wet weight fermented substrate and was scaled up to reflect how much compound would theoretically be produced and extracted from 1 kg of fermented substrate.

## 5. Conclusions

We have screened 36 different fermentations in an exploratory study investigating four filamentous fungi for their potential to produce valuable flavour and aroma metabolites from nine unsupplemented agro-industrial by-products. Hundreds of these volatiles were identified and four compounds of interest were quantified. Preliminary yields are comparatively high when compared to other natural sources. This, combined with the low-cost substrates and high-value products derived from the fermentation process, presents a compelling case to proceed with optimisation of the fermentation parameters and fungal strains to determine if yield and productivity can be further improved. There are also many more candidate compounds that could be further investigated in addition to further stereochemistry determination of targeted compounds. Further screening experiments should focus on substrates that produced the most volatile compounds overall—apple pomace, carrot pomace, and orange pomace—combined with GC analysis on a chiral packed column to report the enantiomeric excess and absolute configuration.

## Figures and Tables

**Table 1:** Potentially industrially relevant volatile compounds produced during fermentation of nine fruit and vegetable by-products using four different filamentous fungi.

Compound	Substrate	Microorganism	Fold-Change ^a^	Descriptor ^b^
1-Butanol	Apple pomace	*A. niger*	3	Oily-banana
2-methyl-1-butanol	Onion pulp	*A. niger*	2	Black truffle
	Onion pulp	*P. camemberti*	191	
	Onion pulp	*P. cinnabarinus*	6	
	Orange pomace	*P. camemberti*	28	
1-Heptanol	Brewer’s spent grain	*A. oryzae*	60	Pleasant, cosmetic
	Brewer’s spent grain	*A. niger*	10	
1-Hepten-3-ol	Onion pulp	*P. camemberti*	165	Oily, green, metallic
1-Hexanol	Brewer’s spent grain	*A. oryzae*	7	Fresh cut grass
1-Octen-3-ol	Brewer’s spent grain	*P. camemberti*	24	Mushroom alcohol
	Brewer’s spent grain	*P. cinnabarinus*	19	
	Kiwifruit peels	*A. oryzae*	31	
	Kiwifruit peels	*A. niger*	29	
	Kiwifruit peels	*P. cinnabarinus*	68	
	Kiwifruit peels	*P. camemberti*	33	
	Onion pulp	*A. niger*	87	
	Onion pulp	*A. oryzae*	38	
	Onion pulp	*P. camemberti*	3118	
	Onion pulp	*P. cinnabarinus*	1555	
6-methyl-2-heptanone	Brewer’s spent grain	*A. oryzae*	54	Camphorous
2-Pentanone	Apple pomace	*P. camemberti*	8	Fruity
3-Octanol	Carrot pomace	*A. oryzae*	2	Mushroom, herbal, citrus
	Carrot pomace	*P. cinnabarinus*	163	
	Brewer’s spent grain	*A. niger*	109	
	Brewer’s spent grain	*P. cinnabarinus*	261	
	Brewer’s spent grain	*P. camemberti*	100	
	Kiwifruit peels	*P. cinnabarinus*	84	
	Kiwifruit peels	*P. camemberti*	5	
	Onion pulp	*A. niger*	423	
	Onion pulp	*A. oryzae*	705	
	Onion pulp	*P. camemberti*	43	
	Onion pulp	*P. cinnabarinus*	182	
	Red grape marc	*A. niger*	48	
	Red grape marc	*A. oryzae*	215	
	White grape marc	*A. niger*	50	
	White grape marc	*A. oryzae*	102	
3-Octanone	Brewer’s spent grain	*A. niger*	26	Herbal, lavender, nectarine
	Brewer’s spent grain	*P. camemberti*	221	
	Brewer’s spent grain	*P. cinnabarinus*	20	
	Kiwifruit peels	*A. oryzae*	9	
	Kiwifruit peels	*A. niger*	4	
	Kiwifruit peels	*P. cinnabarinus*	14	
	Kiwifruit peels	*P. camemberti*	5	
	Onion pulp	*A. niger*	224	
	Onion pulp	*A. oryzae*	193	
	Onion pulp	*P. camemberti*	220	
	Onion pulp	*P. cinnabarinus*	85	
	Red grape marc	*A. oryzae*	58	
	Red grape marc	*A. niger*	30	
	White grape marc	*A. niger*	20	
	White grape marc	*A. oryzae*	25	
Methyl dec-4-enoate	Apple pomace	*P. cinnabarinus*	112	Tropical, fishy
	Carrot pomace	*P. cinnabarinus*	354	
Methyl oct-4-enoate	Carrot pomace	*P. cinnabarinus*	207	Fresh pineapple
Methyl acetate	Apple pomace	*A. niger*	8	Ethereal, sweet, fruity
	Apple pomace	*A. oryzae*	5	
	Carrot pomace	*P. cinnabarinus*	261	
	Carrot pomace	*P. cinnabarinus*	195	
	Orange pomace	*A. niger*	65	
	Orange pomace	*A. oryzae*	14	
α.-Cubebene	Apple pomace	*P. cinnabarinus*	61	Herbal
Amyl isovalerate	Apple pomace	*A. niger*	71	Fruity
Anisole	Brewer’s spent grain	*P. camemberti*	8	Aniseed
	Brewer’s spent grain	*P. cinnabarinus*	786	
	Orange pomace	*A. niger*	21	
	Orange pomace	*P. cinnabarinus*	351	
Benzaldehyde	Brewer’s spent grain	*P. cinnabarinus*	2	Cherry, almond
	Orange pomace	*A. niger*	2	
1,2-dimethoxybenzene	Orange pomace	*A. niger*	92	Insect attractant
1,4-Dimethoxybenzene	Onion pulp	*P. camemberti*	164	Intense sweet, floral
3,4-Dimethylstyrene	Orange pomace	*A. niger*	150	Green, floral, smoky
	Orange pomace	*P. camemberti*	245	
Benzoic acid	Carrot pomace	*P. cinnabarinus*	272	Balsamic
	Kiwifruit peels	*P. cinnabarinus*	29	
	Onion pulp	*P. cinnabarinus*	138	
Methyl benzoate	Orange pomace	*P. camemberti*	16	Feijoa, ylang ylang, wintergreen
	Orange pomace	*P. cinnabarinus*	33	
	Orange pomace	*A. niger*	27	
Benzyl alcohol	Brewer’s spent grain	*P. camemberti*	31	Precursor and solvent
	Brewer’s spent grain	*P. cinnabarinus*	17	
β-Pinene	Kiwifruit peels	*A. niger*	2	Herbal, pine
β-Myrcene	Apple pomace	*A. oryzae*	2	Clove-like
	Kiwifruit peels	*P. camemberti*	2	
Sabinene	Brewer’s spent grain	*P. camemberti*	8	Spicy, black pepper
2-methylbutanal	Brewer’s spent grain	*P. camemberti*	15	Musty, chocolate
	Brewer’s spent grain	*P. cinnabarinus*	13	
3-methylbutanal	Carrot pomace	*P. cinnabarinus*	61	Peach, malty, fatty, chocolate, peach
	Kiwifruit peels	*P. cinnabarinus*	33	
	Kiwifruit peels	*P. camemberti*	4	
	Onion pulp	*A. oryzae*	15	
	Onion pulp	*A. niger*	8	
	Brewer’s spent grain	*A. niger*	11	
	Brewer’s spent grain	*A. oryzae*	10	
	Onion pulp	*A. oryzae*	307	
	Onion pulp	*A. niger*	53	
Ethyl isovalerate	Apple pomace	*A. niger*	210	Fruity
(E)-Cinnemaldehyde	Red grape marc	*A. oryzae*	40	Cinnamon
Cyclopentanone	Kiwifruit peels	*A. oryzae*	37	Minty
	Kiwifruit peels	*A. niger*	17	
Decanal	Olive cake	*P. cinnabarinus*	5	Citrus
(D)-Limonene	Carrot pomace	*A. niger*	6	Citrus
Ethyl tiglate	Red grape marc	*A. niger*	129	Tutti frutti, green olive
Methyl heptanoate	Carrot pomace	*P. cinnabarinus*	106	Fruity, green, waxy
Hexanal	Brewer’s spent grain	*A. oryzae*	3	Fresh cut grass
	Kiwifruit peels	*P. cinnabarinus*	4	
	Olive cake	*P. cinnabarinus*	10	
Methyl hexanoate	Apple pomace	*P. cinnabarinus*	133.85	Pineapple, fatty
	Carrot pomace	*P. cinnabarinus*	75.89	
	Orange pomace	*P. cinnabarinus*	17.40	
Hexyl valerate	Apple pomace	*A. niger*	1.49	Green, brandy
iso-Amyl tiglate	Apple pomace	*A. niger*	21.04	herbal
*trans*-Limonene oxide	Olive cake	*P. cinnabarinus*	36.57	Minty, citrus
Methyl 2-furoate	Apple pomace	*P. cinnabarinus*	130.75	Caramel, musty, fungal
	Carrot pomace	*P. cinnabarinus*	624.14	
	Brewer’s spent grain	*P. camemberti*	11.04	
	Brewer’s spent grain	*P. cinnabarinus*	687.33	
	Kiwifruit peels	*P. cinnabarinus*	588.31	
	Kiwifruit peels	*P. camemberti*	7.72	
	Olive cake	*P. cinnabarinus*	138.46	
	Onion pulp	*P. camemberti*	17.89	
	Onion pulp	*P. cinnabarinus*	816.65	
	Orange pomace	*P. cinnabarinus*	58.68	
Methyl isovalerate	Kiwifruit peels	*P. cinnabarinus*	1294.67	Fruity
	Onion pulp	*P. cinnabarinus*	139.85	
	Red grape marc	*P. cinnabarinus*	395.00	
Methyl salicylate	Orange pomace	*A. niger*	15.49	Wintergreen mint, root beer
	Orange pomace	*P. cinnabarinus*	16.38	
Methyl valerate	Carrot pomace	*P. cinnabarinus*	44.37	Sweet, fruity
Methyleugenol	Apple pomace	*P. camemberti*	845.19	Spicy, clove
	Apple pomace	*P. cinnabarinus*	41.88	
	Orange pomace	*P. camemberti*	48.28	
Methyl nonanoate	Carrot pomace	*P. cinnabarinus*	90.73	Pear, tropical, waxy
Phenyl acetaldehyde	Carrot pomace	*A. niger*	3.15	Honey, floral
	Carrot pomace	*A. oryzae*	1.52	
	Brewer’s spent grain	*A. oryzae*	3.47	
	Brewer’s spent grain	*P. cinnabarinus*	77.04	
	Olive cake	*P. cinnabarinus*	5.73	
Phenylethyl alcohol	Brewer’s spent grain	*P. cinnabarinus*	77.04	Rose
2-methylpropanoate	Carrot pomace	*A. oryzae*	187.92	Rancid butter
	Carrot pomace	*A. niger*	62.03	
2,5-Dimethylpyrazine	Carrot pomace	*A. niger*	3.68	Nutty, musty
	Carrot pomace	*A. oryzae*	2.95	
	Onion pulp	*A. niger*	2.83	
Vanillin	Olive cake	*P. camemberti*	1.14	Vanilla

Filamentous fungi: *Aspergillus niger*, *Aspergillus oryzae*, *Penicillium camemberti*, *and Pycnoporus cinnabarinus*. ^a^ Fold-change is compared to the unfermented negative control of the respective substrate. ^b^ Odour descriptors adapted from George 2005.

**Table 2:** Substrate-specific volatile compounds produced during fermentation of fruit and vegetable by-products using four different filamentous fungi.

Apple Pomace	Brewer’s Spent Grain	Carrot Pomace	Kiwifruit Peels	Olive Cake	Onion Pulp	Orange Pomace	Red Grape Marc
Amyl isovalerate	Benzyl alcohol	2,5-dimethyl-pyrazine	Cyclopentanone	Decanal	1,4-Dimethoxy benzene	Benzoic acid, methyl ester	Cinnemaldehyde, (E)-
1-Butanol	1-Heptanol	Heptanoic acid, methyl ester	β-Pinene	*trans*-Limonene oxide	1-Hepten-3-ol	1,2-Dimethoxy benzene	Ethyl tiglate
α-Cubebene	1-Hexanol	Limonene		Vanillin		3,4-Dimethyl styrene	
Ethyl isovalerate	2-Methyl-butanal	Methyl valerate				Methyl salicylate	
Hexyl n-valerate	6-Methyl-2-heptanone	Nonanoic acid, methyl ester					
iso-Amyl tiglate	Phenylethyl alcohol	4-Octenoic acid, methyl ester, (Z)-					
2-Pentanone	Sabinene	Propanoic acid, 2-methyl-					

Filamentous fungi used: *Aspergillus niger*, *Aspergillus oryzae*, *Penicillium camemberti*, and *Pycnoporus cinnabarinus*.

**Table 3:** Yield of commercially relevant flavour and aroma compounds produced from fermented substrates.

Compound	Value (USD/kg) ^a^	Annual Consumption (kg) ^b^	Substrate	Microorganism	Yield (g/kg) ^c^
Methyl benzoate	USD 335 *	590	Orange	*Aspergillus niger*	0.173 ± 0.0003
Phenylacetaldehyde	USD 450 *	106	SBG	*Pycnorporus cinnabarinus*	1.493 ± 0.384
1-Octen-3-ol	USD 4800 *	250	Onion	*Aspergillus oryzae*	1.297 ± 0.107
Phenylethyl alcohol	USD 500 *	1240	SBG	*Pycnorporus cinnabarinus*	0.970 ± 0.242

^a^ Value per kilogram obtained through personal communication with Jeffrey Buco at Excellentia International. Prices quoted in US dollars correct as of September 2017. ^b^ Annual consumption of compound as a flavour additive only (George, 2005). Excludes other uses, e.g., fragrance and cosmetics industry. ^c^ Yield represents average dry weight of compound produced per kilogram of fermented substrate (wet weight) ± 2 standard deviations (n = 9). * Prices quoted in US dollars correct as of September 2017. Substrates: Orange = orange pomace, SBG = spent brewer’s grain, Onion = onion pulp.

## Data Availability

All data, tables and figures in this manuscript are original and raw data can be obtained by contacting the authors.

## Appendix A

**Table A1:** Calibration curve characteristics for standard aroma metabolites analysed by GC–MS.

Compound	Relative Slope of Regression Line ^a^ (n ≥ 4)	Intercept of Regression Line ^a^ (n ≥ 4)	Coefficient (r^2^)	Linear Range (μM)
Methyl benzoate	13.819	0.0021	>0.99	0.5–7.0
Phenylacetaldehyde	30.800	0.0931	>0.99	10.0–70.0
1-octen-3-ol	30.906	0.0744	>0.99	10.0–70.0
Phenethyl alcohol	33.867	0.1537	>0.99	10.0–70.0

^a^ Regression line: y = m ·x. Slope is reported relative to the slope of internal standard (12-bromo-dodecanol).
